# Risk of preterm delivery and early pregnancy hydroxychloroquine use from a Californian lupus cohort

**DOI:** 10.1136/lupus-2025-001654

**Published:** 2025-09-30

**Authors:** Amadeia Rector, Emily F Liu, Maurice Druzin, Michael H Weisman, Eliza Chakravarty, Miranda Cantu, Gary M Shaw, Daniel Z Kuo, Monique M Hedderson, Julia F Simard

**Affiliations:** 1 Epidemiology and Population Health, Stanford University School of Medicine, Stanford, California, USA; 2 Division of Research, Kaiser Permanente LLC, Oakland, California, USA; 3 Obstetrics and Gynecology, Stanford University School of Medicine, Stanford, California, USA; 4 Medicine, Immunology and Rheumatology, Stanford University School of Medicine, Stanford, California, USA; 5 Arthritis and Clinical Immunology, Oklahoma Medical Research Foundation, Oklahoma City, Oklahoma, USA; 6 Global Lupus Support Group, Portage, Michigan, USA; 7 Stanford University School of Medicine, Stanford, California, USA; 8 Redwood City Medical Center, Kaiser Permanente LLC, Redwood City, California, USA; 9 Division of Research, Kaiser Permanente Northern California, Oakland, California, USA

**Keywords:** Lupus Erythematosus, Systemic, Epidemiology, Health services research

## Abstract

**Objective:**

Pregnant patients with systemic lupus erythematosus (SLE) have 2-3 times higher risk of preterm delivery (PTD). Hydroxychloroquine (HCQ) is recommended during pregnancy and may reduce PTD risk. This study investigates whether early pregnancy HCQ-use reduces PTD risk in a diverse SLE cohort.

**Methods:**

We included singleton pregnancies reaching ≥20 weeks’ gestation (2011–2020) among patients with SLE aged 18–50 receiving care at Kaiser Permanente Northern California. HCQ exposure was defined as ≥2 prescriptions filled from 3 months before the last menstrual period through the first trimester. PTD was defined as delivery <37 weeks and continuously as gestational weeks for time-to-delivery analyses. Propensity scores (PS) based on demographics, comorbidities and medication use were calculated to address confounding. Risk ratios (RR) and HRs, including 95% CIs, were estimated using PS-adjusted Poisson regression with robust SEs and Cox regression, stratified by parity. To investigate effect modification, we stratified by prepregnancy comorbidities and pregnancy corticosteroid use.

**Results:**

Among 399 pregnancies in 324 patients, 21% were preterm. The PS-adjusted RR was 1.08 (95% CI 0.52 to 2.23) and 0.88 (95% CI 0.50 to 1.57) for nulliparous and multiparous pregnancies exposed to HCQ, respectively. The PS-adjusted HRs were similar, and results remained consistent across analyses stratified by potential effect modifiers.

**Conclusions:**

Although periconceptional HCQ-use was not associated with reduced PTD nor appreciably altered gestational age at delivery, we found no increased risks for these specific adverse outcomes. Consistent with other work, we found potentially protective associations in subsets stratified by parity. However, we had limited statistical power to test this.

**WHAT IS ALREADY KNOWN ON THIS TOPIC**

**WHAT THIS STUDY ADDS**

- Using a large, diverse cohort of pregnancies among patients with systemic lupus erythematosus, we found no association between HCQ exposure in early pregnancy and preterm delivery despite addressing the numerous methodologic limitations in previous work and extensive sensitivity analyses. Consistent with other work, we found potentially protective associations in subsets stratified by parity. However, we had limited power to test this.

**HOW THIS STUDY MIGHT AFFECT RESEARCH, PRACTICE OR POLICY**

- We found no evidence of harm or risk related to preterm delivery associated with HCQ use in early pregnancy.

## Introduction

 Systemic lupus erythematosus (SLE) is an autoimmune rheumatic disease predominantly affecting females of childbearing age and has 2–3 times higher risk for preterm delivery (PTD) compared with the general population.^1 2^ Risk factors have been identified that predispose patients with SLE to PTD; they include active lupus before pregnancy, flares during pregnancy, history of lupus nephritis, chronic hypertension, damage and pre-eclampsia or eclampsia, which may lead to medically indicated early delivery.^3^,^5^ Up to 60%–80% of pre-eclampsia cases result in medically indicated PTD among patients with SLE.^6^,^8^ Flares are a risk factor for both pre-eclampsia and PTD, and the use of hydroxychloroquine (HCQ) in pregnancy may prevent SLE flares.^9 10^ The discontinuation of HCQ, an agent which is a mainstay for SLE treatment, in the 3 months before pregnancy, during the first trimester, or not taken throughout pregnancy, has also been linked to pre-eclampsia and PTD.^11^,^13^

HCQ is considered generally safe for use in pregnancy and is one of few drugs recommended to manage SLE before and during pregnancy by the European Alliance of Associations for Rheumatology and the American College of Rheumatology.^14 15^ While extensive evidence suggests that HCQ likely does not pose a significant teratogenic risk in pregnancy, the Teratogen Information System notes that current data remain insufficient to confirm its complete safety.^1316^,^21^ Research suggests that HCQ may reduce reactive oxygen species and tumour necrosis factor-α (TNF-α)—key contributors to oxidative stress and inflammation linked to placental dysfunction—though the exact mechanism is unknown.^22^ In preeclampsia, placental dysfunction may result from elevated proinflammatory cytokines, such as TNF-α, triggered by placental ischaemia and oxidative stress-induced endothelial dysfunction, potentially leading to medically indicated PTD. Since inflammation is also implicated in spontaneous PTD, HCQ presents a promising preventive option for both medically indicated and spontaneous PTD.^23^ Some small observational studies support the use of HCQ in pregnancy to prevent PTD and preeclampsia,^24^,^27^ although results are inconsistent.^2028^,^30^ Additionally, a small randomised controlled trial and several observational studies demonstrated that HCQ exposure in pregnancy prolongs gestational duration.^13 26 31^ Despite the safety of HCQ and its recommended use in pregnancy through various institutional guidelines, use during pregnancy in the USA is only 40%–50% among patients with lupus.^7 29^

Many of the epidemiological studies examining the association between HCQ in pregnancy and PTD were underpowered, without appropriate comparators (ie, no indication for HCQ, differing PTD risk profile), did not consider bias due to confounding and did not stratify by parity. Using a large, diverse cohort of pregnancies among patients with SLE, we investigated the association between HCQ exposure in early pregnancy and PTD overall and its subtypes.

## Methods

### Data source

Kaiser Permanente Northern California (KPNC) is an integrated healthcare delivery organisation providing comprehensive medical care that covers approximately 4 million diverse members from 16 counties. All care is captured in the electronic health records (EHR) and includes inpatient, emergency and outpatient claims, laboratory orders and results, procedures and prescription orders and fills.^32^

The perinatal obstetric database of all pregnancies in KPNC was used to identify this cohort. Pregnancies ending in live births are linked to an established infant cohort.^33^ The obstetric database consolidates data across multiple sources, has several quality control checks and includes information on prepregnancy care and comorbidities, medication use, hospitalisation, delivery admission and postpartum care.

### Study population

This study includes singleton deliveries among patients with a prevalent SLE diagnosis aged 18–50 years who received care from 2011 to 2020 from KPNC. Prevalent SLE was defined as ≥2 ICD-coded inpatient or outpatient visits (ICD-9: 710.0; ICD-10: M32.1*, M32.8, M32.9) ≥7 days apart, and we restricted to pregnancies satisfying this definition at or before the patient’s estimated last menstrual period (LMP). The LMP was defined using estimated delivery date, which was calculated using the infant’s birth date and gestational age (GA). We restricted our cohort to pregnancies that completed at least 20 weeks’ gestation to include both stillbirth and live birth deliveries and excluded pregnancies lacking parity data (n=17). While some similarities in age, neighbourhood deprivation indices and history of nephritis were noted between the included and excluded, the excluded group had a lower proportion of pregnancies to white patients, more were publicly insured, and fewer had prepregnancy hypertension identified.

### Exposure

**Figure 1:**
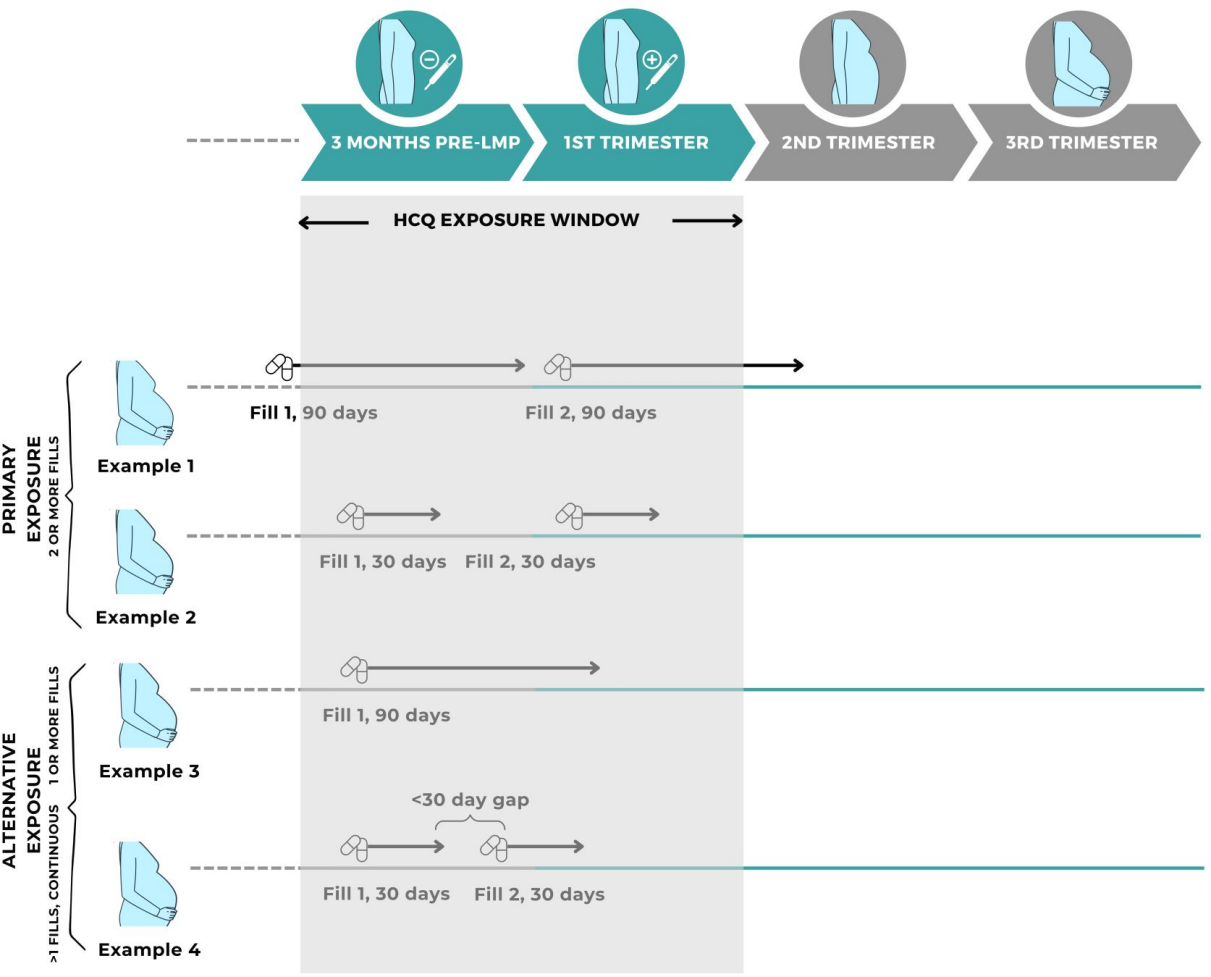
Hydroxychloroquine exposure window in pre and early pregnancy. The hydroxychloroquine exposure window is defined as 3 months pre-LMP to the end of the first trimester. Examples 1 and 2 are examples of primary exposure, consisting of two fills in or covering the exposure window. Example 3 consists of one fill in the exposure window, and example 4 consists of two fills in the exposure window with <30 days gap between fills. Icons taken from Alphavector and slebor via Canva.com. HCQ, hydroxychloroquine; LMP, last menstrual period.

HCQ exposure was primarily defined as ≥2 medication fills occurring during the exposure window—3 months before LMP through the end of the first trimester. This definition includes fills occurring prior to and carrying over into the exposure window (eg, a 90-day supply fill occurring 4 months before LMP would contribute). Pregnancies were considered unexposed if there was no carry over nor fills during the exposure window. In sensitivity analyses, we alternatively defined HCQ exposure two ways: (1) ≥1 fills occurring during the exposure window (including carry over) given 64% of fills cover 100 days, which would be a sufficiently long exposure; (2) continuous exposure defined as ≥2 fills with gaps in fill coverage <30 days to account for potential discontinuation, which is not accounted for in the primary exposure definition (figure 1). The varying definitions of HCQ exposure in early pregnancy should be sufficient given its long half-life of approximately 40 days even considering altered metabolism in pregnancy.^34^,^36^

### Outcome

PTD was defined as delivery occurring at less than 37 weeks GA and also further subcategorised by indication—spontaneous, medically indicated or unknown—and categorically by GA—extremely preterm (<28 weeks GA), very preterm (28 to <32 weeks GA) and moderately preterm (32 to <37 weeks GA). PTD was designated as spontaneous if there was an ICD code for early spontaneous onset of delivery (ICD-9: 644.20, 644.21; ICD-10: O60.1*) and medically indicated if there was no ICD code for early spontaneous onset of delivery and a record of caesarean delivery or induction of labour in the index pregnancy.^37^ For the time-to-delivery analyses, PTD was defined continuously as the number of weeks of gestation up to 37 weeks.

### Comorbidities and other covariates

We extracted demographics, comorbidities, filled medications and pregnancy characteristics from the KPNC medical records and pregnancy database (definitions included in online supplemental table 1).

Demographic information included maternal age (age at LMP), self-reported race/ethnicity, neighbourhood deprivation index (NDI) and insurance status (public vs commercial). Patients’ residential addresses were geocoded and linked to USA census tracts to calculate the NDI, a multifactor variable encompassing socioeconomic status and disparity derived from the American Community Survey (ACS) data.^38^ The NDI was calculated using the entire KPNC patient population with year-specific cut-offs corresponding to ACS data, assigned to pregnancies based on delivery date and categorised into quintiles of increasing deprivation.

Comorbidities and SLE disease characteristics included prepregnancy body mass index (BMI), prepregnancy hypertension, diabetes, history of lupus nephritis and antiphospholipid antibodies status (aPL). Positive aPL status (anticardiolipin antibodies, anti-beta2 glycoprotein 1 antibodies and lupus anticoagulant) was extracted from lab results and defined as a history of titre >40 any time before delivery.

Medication use was collected for non-fluorinated corticosteroids (oral, injection and intravenous) taken before pregnancy (≥1 fills or administrations 6 months pre-LMP) and azathioprine, non-fluorinated corticosteroids, heparin and labetalol taken during pregnancy (≥1 fills or administrations). Corticosteroid and azathioprine use were considered markers of active disease before and during pregnancy.

Pregnancy characteristics and outcomes included pre-eclampsia, preterm premature rupture of membranes (PPROM), prepregnancy hypertension and gestational diabetes. Pre-eclampsia was defined by ICD codes for pre-eclampsia and eclampsia. Parity, categorised as nulliparous or multiparous, was derived from medical record notes spanning pregnancy onset through 90 days after pregnancy end date. Data on prior pre-eclampsia and prior PTD were extracted for multiparous pregnancies given the increased risk of subsequent PTD. A multiparous pregnancy was classified as having prior PTD and/or prior pre-eclampsia if there was at least one prior pregnancy recorded within the KPNC pregnancy database, resulting in PTD and/or diagnosed with pre-eclampsia, respectively. Those with an incomplete pregnancy history (ie, discordance between the number of patient-reported past pregnancies and pregnancies recorded in the KPNC pregnancy database) were considered to have unknown status for prior PTD (n=39) and prior pre-eclampsia (n=41). Otherwise, the pregnancy was considered to have no prior PTD and/or prior pre-eclampsia.

### Statistical analyses

We described demographics, comorbidities, medication use and pregnancy characteristics and outcomes as frequencies and median values (as indicated) stratified by HCQ exposure, as defined in primary and sensitivity analyses. All analyses were stratified by parity.

### PTD versus term delivery

We calculated propensity scores (PS) separately among nulliparous and multiparous pregnancies and included covariates we believed to be potential confounders based on subject matter knowledge and prior work.^30^ The covariates included maternal age, prepregnancy BMI (categorised as normal, underweight, overweight and obese), race/ethnicity, NDI, diabetes, prepregnancy hypertension, history of lupus nephritis, prepregnancy corticosteroid use, azathioprine use in pregnancy and aPL status. For multiparous pregnancies, the PS additionally included prior PTD and prior pre-eclampsia. We trimmed the datasets based on the area of common support to account for non-positivity. Modified Poisson models estimated crude and PS-adjusted risk ratios (RR) and 95% CIs for PTD comparing pregnancies exposed to HCQ in early pregnancy to pregnancies unexposed to HCQ. In analyses among multiparous pregnancies, robust SEs accounted for autocorrelation (ie, multiple deliveries by the same mother).

### Time-to-PTD

We calculated crude and PS-adjusted HRs and 95% CIs for time-to-PTD comparing pregnancies exposed to HCQ in early pregnancy against pregnancies unexposed to HCQ using Cox regression with GA (measured in weeks) as underlying time. Time zero was set at 19 weeks GA (given that pregnancies resulting in deliveries must make it to 20 weeks gestation) and individuals contributed person-time until PTD or 37 weeks (term). The proportional hazards assumption was assessed by visually examining Kaplan-Meier survival curves and log–log plots and analysing Schoenfeld residual tests for the interaction of time with covariates (HCQ exposure and PS).^39 40^

For the above models, we performed PS-adjusted stratified analyses to assess for effect modification by prepregnancy hypertension, history of lupus nephritis, aPL status and corticosteroid use during pregnancy (as a proxy for flare).

Sensitivity analyses evaluated the secondary definitions of HCQ exposure: ≥1 fills versus 0 fills; and ≥2 fills continuously filled versus 0 fills.

We used R V.4.3.1 (2023.09.1+494) for data management and statistical analyses. The study team included patient partners from the study design phase, through the analysis and interpretation of this work, and drafting of the final manuscript.

### Patient and public involvement

The study team included patient partners from the study design phase, through the analysis and interpretation of this work and drafting of the final manuscript.

## Results

We observed 477 pregnancies among 375 patients with SLE who delivered at KPNC between 2011 and 2020, 399 of which (from 324 patients with SLE) were included in the primary analysis defining HCQ exposure as at least two fills. The median maternal age was approximately 33 years, and the patients came from many different race and ethnic groups (table 1).

**Table 1:** Demographics and clinical characteristics for singleton deliveries (live and stillbirth) in patients with SLE by hydroxychloroquine (HCQ) exposure (≥2 fills vs 0 fills) and parity, presented as n (%) unless otherwise specified

Characteristics	All pregnancies	Nulliparous pregnancies		Multiparous pregnancies	
		HCQ+	HCQ−	HCQ+	HCQ−
	n=399	n=85	n=78	n=94	n=142
**Demographic characteristics**					
Maternal age in years, median (IQR)*	32.7 (29.5, 35.5)	31.2 (28.8, 34.9)	31.0 (26.8, 33.4)	33.2 (30.0, 36.0)	33.9 (30.9, 36.0)
Race/ethnicity					
Asian	118 (29.6)	33 (38.8)	22 (28.2)	32 (34.0)	31 (21.8)
Black	36 (9.0)	7 (8.2)	7 (9.0)	8 (8.5)	14 (9.9)
Hispanic	90 (22.6)	16 (18.8)	22 (28.2)	19 (20.2)	33 (23.2)
Other†	36 (9.0)	9 (10.6)	6 (7.7)	8 (8.5)	13 (9.2)
White	119 (29.8)	20 (23.5)	21 (26.9)	27 (28.7)	51 (35.9)
Neighbourhood deprivation index (residence at delivery)‡					
Quintile 1 (least deprived)	31 (10)	6 (9.5)	<5	9 (15)	12 (10)
Quintile 2	56 (19)	10 (16)	16 (27)	14 (23)	16 (14)
Quintile 3	84 (28)	17 (27)	16 (27)	12 (19)	39 (34)
Quintile 4	72 (24)	15 (24)	12 (20)	13 (21)	32 (28)
Quintile 5 (most deprived)	58 (19)	15 (24)	12 (20)	14 (23)	17 (15)
Public insurance	57 (14)	11 (13)	14 (18)	12 (13)	20 (14)
**Maternal morbidities and clinical characteristics**					
History of lupus nephritis	80 (20)	19 (22)	15 (19)	22 (23)	24 (17)
Positive aPL lab§	74 (19)	21 (25)	16 (22)	14 (15)	23 (18)
Prepregnancy hypertension	65 (16)	11 (13)	17 (22)	19 (20)	18 (13)
Prepregnancy BMI, median (IQR)	24.9 (21.8, 29.6)	24.7 (21.6, 29.2)	25.3 (22.1, 28.6)	24.6 (21.7, 31.1)	25.0 (22.0, 29.7)
**Pregnancy complications**					
Gestational hypertension	100 (25)	28 (33)	22 (28)	18 (19)	32 (23)
Preeclampsia	71 (18)	20 (24)	16 (21)	13 (14)	22 (16)
Early onset pre-eclampsia (<34 weeks GA)	22 (6)	5 (6)	6 (8)	5 (5)	6 (4)
PPROM	18 (5)	<5	<5	6 (6)	5 (4)
Gestational diabetes	16 (4)	<5	<5	<5	10 (7)
**Prior pregnancy history**					
Prior preterm (<37 weeks GA)				31 (36)	43 (36)
Prior pre-eclampsia				20 (24)	37 (32)
**Medications**					
Corticosteroids¶	92 (23)	33 (39)	13 (17)	30 (32)	16 (11)
Azathioprine**	39 (10)	15 (18)	7 (9)	10 (11)	7 (5)
Heparin**	28 (7)	7 (8)	6 (8)	7 (7)	8 (6)
Labetalol**	47 (12)	13 (15)	12 (15)	11 (12)	11 (8)

* Maternal age at estimated date of last menstrual period (LMP).

† Other race and ethnicity category includes multiracial, Native American, Pacific Islander and unknown.

‡ Neighbourhood deprivation index n=98 (25%) missing.

§ aPL n= 25 (6%) missing.

¶ 6 months pre-LMP through LMP.

** Pregnancy onset through delivery.

aPL, antiphospholipid antibodies; BMI, body mass index; GA, gestational age; PPROM, preterm premature rupture of membranes.

Overall, 38% of pregnancies had at least two HCQ fills in early pregnancy—nulliparous pregnancies were more likely to be exposed (45%) compared with multiparous pregnancies (33%). Among the 179 pregnancies with at least two HCQ fills, 98% continued filling prescriptions into the second or third trimester. HCQ exposure increased to 54% when defined as at least one fill and dropped to 19% when defined as two or more continuous fills occurring during or overlapping with the exposure window. A small percentage of pregnancies initiated HCQ in the second (5%) and third trimesters (2%) (online supplemental table 2). Among HCQ-exposed pregnancies, the median estimated dose was 300 mg/day (IQR: 200–400 mg/day).

HCQ-exposed nulliparous pregnancies had a lower prevalence of prepregnancy hypertension compared with HCQ-unexposed (13% vs 22%), whereas the opposite was observed among multiparous pregnancies (20% vs 13%). Among all HCQ-exposed pregnancies, there was a higher prevalence of past lupus nephritis, and more prepregnancy corticosteroid use and azathioprine use during pregnancy. Few pregnancies (n=6) had pre-pregnancy diabetes (table 1, online supplemental tables 3 and 4).

Almost 20% (n=71) of pregnancies were complicated by pre-eclampsia with more than half (38/71) delivered preterm. Nulliparous pregnancies experienced more pre-eclampsia while multiparous pregnancies experienced less. Gestational hypertension was common (25%), while gestational diabetes (4%) and PPROM (5%) were less common. Among multiparous pregnancies, 36% had a history of PTD, and prior pre-eclampsia was less common among the exposed (24% vs 32%) (table 1, online supplemental tables 3 and 4).

Twenty-one per cent of pregnancies were delivered preterm and most were medically indicated (64%). The majority of preterm deliveries (76%) occurred between 32 and <37 weeks’ gestation, while 13% occurred between 28 and <32 weeks, and 11% before 28 weeks. Crude PTD risk was slightly higher among nulliparous HCQ-exposed pregnancies (20%, n=17) compared with unexposed pregnancies (17%, n=20), whereas the opposite was observed for multiparous pregnancies (21%, n=20 HCQ-exposed vs 23%, n=33 HCQ-unexposed). Among multiparous pregnancies, HCQ-exposed pregnancies resulted in fewer medically indicated preterm deliveries (11% vs 17%) (table 2). The median GA at delivery was slightly higher among the exposed (35 weeks vs 34 weeks among HCQ-unexposed) (table 2, online supplemental tables 5 and 6).

**Table 2:** Pregnancy delivery outcomes and features by hydroxychloroquine (HCQ) exposure (≥2 fills vs 0 fills) and parity, presented as n (%) unless otherwise specified

Characteristics	All pregnancies	Nulliparous pregnancies		Multiparous pregnancies	
		HCQ+	HCQ−	HCQ+	HCQ−
	n=399	n=85	n=78	n=94	n=142
**Delivery outcomes and features**					
Gestational age at delivery, median (IQR)	38 (37, 39)	38 (37, 39)	38 (37, 39)	38 (37, 39)	39 (37, 39)
Gestational age at delivery among preterm deliveries, median (IQR)*	35 (32, 36)	35 (34, 35)	34 (30, 34)	35 (32, 36)	34 (33, 36)
Preterm (<37 weeks GA)	83 (21)	17 (20)	13 (17)	20 (21)	33 (23)
Medically indicated*	53 (64)	11 (65)	8 (62)	10 (50)	24 (73)
Spontaneous*	26 (31)	6 (35)	<5	10 (50)	7 (21)
Moderately preterm (32 to <37 weeks GA)*	63 (76)	14 (82)	8 (62)	15 (75)	26 (79)
Caesarean section†	148 (37)	31 (37)	30 (39)	38 (40)	49 (35)
Stillbirths	5 (1)	<5	<5	<5	<5

* Denominator is preterm deliveries.

† Caesarean section 17 (4%) missing.

GA, gestational age.

We observed no clear association between PTD and HCQ exposure in our main analyses nor sensitivity analyses where HCQ exposure was alternatively defined. The PS-adjusted RR was 1.08 (95% CI 0.52 to 2.23) and 0.88 (95% CI 0.50 to 1.57) among nulliparous and multiparous pregnancies, respectively. Results were similar when looking at time to PTD. Among multiparous pregnancies, HCQ exposure appeared to decrease the risk and rate of PTD in most PS-adjusted analyses stratified by potential effect modifiers except for those with corticosteroid use during pregnancy (RR=1.29, 95% CI 0.35 to 1.66) (tables3  4).

**Table 3:** The association between preterm delivery and early pregnancy hydroxychloroquine (HCQ) exposure (≥2 fills vs 0 fills) estimated by modified Poisson regression among patients with SLE stratified by parity

Analyses	Nulliparous pregnancies		Multiparous pregnancies	
	n*	RR (95% CI)	n*	RR (95% CI)
Crude	146	1.12 (0.57 to 2.18)	193	0.89 (0.52 to 1.52)
PS-adjusted†	146	1.08 (0.52 to 2.23)	193	0.88 (0.50 to 1.57)
**PS-adjusted stratified**				
Prepregnancy hypertension				
Yes	26	1.26 (0.55 to 2.89)	32	0.99 (0.31 to 3.16)
No	120	1.00 (0.34 to 2.92)	161	0.77 (0.39 to 1.52)
History of lupus nephritis				
Yes	30	1.55 (0.42 to 5.78)	38	0.81 (0.46 to 1.43)
No	116	1.03 (0.40 to 2.62)	155	0.96 (0.49 to 1.88)
aPL positive				
Yes	34‡	1.16 (0.27 to 5.03)	33§	0.76 (0.35 to 1.66)
No	108‡	1.12 (0.48 to 2.61)	158§	0.83 (0.45 to 1.53)
Corticosteroid use during pregnancy				
Yes	38	0.87 (0.33 to 2.25)	37	1.29 (0.46 to 3.58)
No	108	1.28 (0.51 to 3.24)	156	0.76 (0.35 to 1.66)

* Sample size after trimming dataset based on area of common support.

† The propensity score included covariates maternal age, prepregnancy BMI, maternal race/ethnicity, neighbourhood deprivation index, diabetes, prepregnancy hypertension, history of lupus nephritis, prepregnancy corticosteroid use, azathioprine use during pregnancy and aPL positive status. Prior preterm birth and prior preeclampsia were included for multiparous pregnancies.

‡ Missing aPL positive status for four nulliparous pregnancies.

§ Missing aPL positive status for two multiparous pregnancies.

aPL, antiphospholipid antibodies; BMI, body mass index; PS, propensity score.

**Table 4:** The association between time to preterm delivery and early pregnancy hydroxychloroquine (HCQ) exposure (≥2 fills vs 0 fills) estimated by Cox regression among patients with SLE stratified by parity

Analyses	Nulliparous pregnancies		Multiparous pregnancies	
	n*	HR (95% CI)	n*	HR (95% CI)
Crude	146	1.10 (0.52 to 2.30)	193	0.83 (0.45 to 1.54)
PS-adjusted†	146	1.03 (0.46 to 2.29)	193	0.81 (0.41 to 1.58)
**PS-adjusted stratified**				
Prepregnancy hypertension				
Yes	26	1.18 (0.38 to 3.74)	32	1.01 (0.26 to 3.99)
No	120	0.99 (0.34 to 2.92)	161	0.74 (0.34 to 1.62)
History of lupus nephritis				
Yes	30	1.73 (0.33 to 8.99)	38	0.52 (0.14 to 1.94)
No	116	0.98 (0.37 to 2.63)	155	0.95 (0.44 to 2.05)
aPL positive				
Yes	34‡	1.11 (0.21 to 5.81)	33§	0.54 (0.09 to 3.24)
No	108‡	1.08 (0.43 to 2.71)	158§	0.81 (0.38 to 1.70)
Corticosteroid use during pregnancy				
Yes	38	0.76 (0.23 to 2.45)	37	0.97 (0.30 to 3.12)
No	108	1.28 (0.45 to 3.64)	156	0.72 (0.30 to 1.72)

* Sample size after trimming dataset based on area of common support.

† The propensity score included covariates maternal age, prepregnancy BMI, maternal race/ethnicity, neighbourhood deprivation index, diabetes, prepregnancy hypertension, history of lupus nephritis, prepregnancy corticosteroid use, azathioprine use during pregnancy and aPL positive status. Prior preterm birth and prior preeclampsia were included for multiparous pregnancies.

‡ Missing aPL positive status for four nulliparous pregnancies.

§ Missing aPL positive status for two multiparous pregnancies.

aPL, antiphospholipid antibodies; BMI, body mass index; PS, propensity score.

In sensitivity analyses, results were qualitatively similar though we saw some fluctuations in PS-adjusted RRs and HRs (online supplemental tables 3–8).

## Discussion

HCQ exposure in early pregnancy was not associated with risk or rate of PTD in lupus pregnancies. Our findings were consistent across multiple sensitivity analyses, including redefining HCQ exposure to include single fills or continuous exposure (≥2 fills, gaps in fill coverage <30 days). Despite clinical recommendations, which overlapped with the study period, the proportion of pregnancies exposed to HCQ was relatively low. This is consistent with other work in the US and abroad.^17 29 41 42^

Our findings are consistent with some, but not all, studies on HCQ use in pregnancy. While one recent pooled meta-analysis of prospectively collected lupus pregnancies found that pregnancies exposed to HCQ had lower odds of high disease activity yet no association with PTD (OR=0.68, 95% 0.39 to 1.18),^9^ another meta-analysis concluded that the odds of PTD were lower in pregnancies among patients with SLE exposed to HCQ (OR=0.63, 95% CI 0.48 to 0.82, I^2^=27.63%).^43^ A recent prospective study measuring HCQ serum levels found no association between APO including PTD and early pregnancy HCQ serum levels >500 ng/mL (therapeutic) or >200 ng/mL (adherence) in univariable analyses. PTD due to placental insufficiency was descriptively lower in the therapeutic group (14.3% vs 23.5%).^36^ Moreover, a study by Nguyen *et al* leveraging Swedish register data employing methods similar to our study concluded that early pregnancy HCQ use was not associated with PTD overall, but potentially with lower odds of medically indicated preterm versus term delivery (OR=0.44, 95% CI 0.19 to 1.01) among multiparous pregnancies.^44^ In our study, we similarly observed a trend towards a protective association among multiparous pregnancies, but the estimated risk reduction (12%) was weaker and the CIs were wide (RR=0.88, 95% CI 0.50 to 1.57) due to limited statistical power. Additionally, our findings of a slightly longer gestation among HCQ exposed pregnancies delivered preterm (median 35 vs 34 weeks) are consistent with another study by Canti *et al.* This study observed prolonged time to delivery among HCQ-exposed pregnancies (37.8±1.72 vs 36.3±4.11 gestational weeks).^45^

The study population was comparable to other cohorts in the USA and Europe though more racially and ethnically diverse than several European cohorts.^6 29 46^ The frequency of pre-eclampsia and PTD among patients with lupus was similar to cohorts in North America and Asia, but slightly higher than in Europe, which may also be due to methodologic differences between studies (notably restricting to deliveries vs all pregnancies and grouping SLE with other autoimmune rheumatic diseases).^6 8 10 24 26 27 29 47 48^

HCQ discontinuation or abstinence in pregnancy increases the risk of severe flare and consequently the risk of adverse pregnancy outcomes.^8 11 18 36^ Compared with several cohorts from tertiary care referral centres, we observed less HCQ treatment in pregnancy, including less HCQ use in early pregnancy.^6 10 27 49^ However, these frequencies align with USA insurance claims data utilising the same definition of ≥2 HCQ fills and Swedish register data.^17 29^ As expected, pregnancies exposed to HCQ tended to have a higher burden of disease including history of nephritis and recent medication use (corticosteroids, azathioprine) and were, therefore, at higher risk for PTD. This may have biased estimates towards increased risks, if residual confounding persisted. It is also worth noting that during the early months of the COVID-19 pandemic (March–April 2020), a surge in HCQ prescribing led to some disruptions for patients with SLE.^50^ However, because only a small proportion of our sample falls within this time period, any impact on our findings is likely minimal.

As an observational retrospective study, there are going to be limitations, some that are due to features of the data and others that are methodological. A claims-based definition of SLE may have introduced some misclassification, the largest concern being that individuals without SLE were included and labelled as SLE pregnancy, and that we would expect that those false positives would have a lower underlying risk of adverse pregnancy outcomes and be unlikely to be prescribed HCQ. This would artificially decrease the risk of PTD in the unexposed group, which could underestimate the potential protective effect of treatment. In addition, the HCQ-unexposed group may include individuals with low-activity or controlled SLE who were not prescribed HCQ, those who were prescribed HCQ but did not fill it, or others with misclassified disease or with a contraindication to HCQ. The potential heterogeneity of the HCQ-unexposed group may reflect lower overall disease activity, which could bias results towards the null and underestimate the true effect of HCQ. However, the majority of patients included had a documented positive antinuclear antibody test (87%) in addition to multiple SLE-coded healthcare visits, and similar algorithms for identifying SLE have been previously validated and used in administrative and claims data.^29 51 52^ Unfortunately, data on disease damage or antibody testing prior to enrolling in KPNC for their care were unavailable.

Several factors of interest were either missing or unavailable to us including alcohol, smoking and aspirin use in pregnancy. While low-dose aspirin is a recommended medication for preventing pre-eclampsia in high-risk populations, we were unable to track its use since it is primarily obtained over-the-counter.^53^ Smoking is associated with increased SLE disease activity, renal involvement and cumulative organ damage, all of which may increase the risk of PTD.^54 55^ However, due to high missingness in smoking data, we were unable to include it in our analyses. We relied on corticosteroid and azathioprine use in pregnancy and prepregnancy as proxies for disease activity and flare. We may be overestimating disease activity by capturing any corticosteroid and azathioprine use as opposed to initiation or escalation of these therapies, which could affect the precision of our results when adjusting for corticosteroid use. Patients may use these medications for maintenance therapy rather than solely for treating an active flare; however, this may indicate ongoing or recent elevated disease activity. To address these pitfalls, we used rigorous methods including propensity score adjustment to reduce residual confounding and confounding by indication. It is possible that there may have been misclassification of HCQ exposure relying on prescription fill data; however, we conservatively defined HCQ exposure as at least two fills in early pregnancy and incorporated sensitivity analyses with varying HCQ exposure definitions.

There are important strengths attributed to this study. We employed a large, EHR-based cohort of individuals in the KPNC system who receive virtually all of their care within the system, which greatly reduces missing data; this is unlike data from other USA tertiary care centres, although differences in other healthcare settings may yield different results. Our racially and ethnically diverse cohort had similar demographics to pregnancies among patients with SLE delivered in California.^48^ Serological data allowed for improved characterisation of aPL positivity, which is often unavailable in claims data and related to adverse pregnancy outcomes. We used rigorous methods such as PS to address confounding by indication and stratified by parity, which is often ignored in analyses despite the varying risk of pregnancy complications and outcomes.^56^

## Conclusion

This study found no association between early pregnancy HCQ use and PTD in lupus pregnancies. The small size of this cohort after stratifying by parity and any residual and unmeasured confounding may have obscured a potential protective effect, as has been observed in some, but not all, studies. Further research into whether HCQ impacts PTD via population-based studies and prospective pregnancy cohorts among patients with autoimmune rheumatic disease need to be performed and clearly would enhance validity and understanding of this important subject.

## Supplementary material

10.1136/lupus-2025-001654 online supplemental file 1

## Data Availability

Data may be obtained from a third party and are not publicly available.
