# Supplementary material for: Risk of preterm delivery and early pregnancy hydroxychloroquine use from a Californian lupus cohort
Source: Lupus Sci Med. 2025 Sep 30;12(2):e001654. doi: 10.1136/lupus-2025-001654 (PMC12481301; doi:10.1136/lupus-2025-001654)
Supplement: online supplemental file 1 [file lupus-12-2-s001.docx]

| **Table S1** Definition and coding of maternal complications and comorbidities | |
| --- | --- |
| **Condition** | **Code** |
| Preeclampsia | **ICD9: 642.4*, 642.5*:** 642.4, 642.40, 642.41, 642.42, 642.43, 642.44, 642.5, 642.50, 642.51, 642.52, 642.53, 642.54  **ICD10: O14.*:**  O14, O14.0, O14.00, O14.02, O14.03, O14.04, O14.05, O14.1, O14.10, O14.12, O14.13, O14.14, O14.15, O14.2, O14.20, O14.22, O14.23, O14.24, O14.25, O14.9, O14.90, O14.92, O14.93, O14.94, O14.95 |
| Eclampsia | **ICD9:** 642.6, 642.60, 642.61, 642.62, 642.63, 642.64, 642.70, 642.71, 642.72, 642.73, 642.74  **ICD10: O15.*:**  O15.00, O15.02, O15.03, O15.1, O15.2, O15.9 |
| Preterm premature rupture of membranes (PPROM) | **ICD9:**  642.4*, 642.5*, 642.6*, 642.7*  **ICD10:**  O14.*, O15.*, O11.* |
| History of lupus nephritis | **ICD9:**  580.*, 581.*, 582.*, 583.*, 584.*, 585.*, 586, 791.0  **ICD10:**  M32.14, N00.*, N01.*, N02.2, N03.*, N04.*, N05.*, N06.*, N08, N17.*, N18.*, N19, R80.9 |
| Pre-pregnancy hypertension | **ICD9: 401*-405*:**  401, 401.0, 401.1, 401.9, 402, 402.0, 402.00, 402.01, 402.1, 402.10, 402.11, 402.9, 402.90, 402.91, 403, 403.0, 403.00, 403.01, 403.1, 403.10, 403.11, 403.9, 403.90, 403.91, 404, 404.0, 404.00, 404.01, 404.02, 404.03, 404.10, 404.11, 404.12, 404.13, 404.9, 404.90, 404.91, 404.92, 404.93, 405, 405.0, 405.01, 405.09, 405.1, 405.11, 405.19, 405.9, 405.91, 405.99  **ICD10: I10-I15 (excluding I14):**  I10, I11, I11.0, I11.9, I12, I12.0, I12.9, I13, I13.0, I13.1, I13.10, I13.11, I13.2, I15, I15.0, I15.1, I15.2, I15.8, I15.9 |
| Diabetes mellitus | **ICD-9-CM:** 250.xx.  **ICD-10-CM:** E10.10, E10.11, E10.21, E10.22, E10.29, E10.311, E10.319, E10.321, E10.329, E10.331, E10.339, E10.341, E10.349, E10.351, E10.359, E10.36, E10.39, E10.40, E10.41, E10.42, E10.43, E10.44, E10.49, E10.51, E10.52, E10.59, E10.610, E10.618, E10.620, E10.621, E10.622, E10.628, E10.630, E10.638, E10.641, E10.649, E10.65, E10.69, E10.8, E10.9, E11.00, E11.01, E11.21, E11.22, E11.29, E11.311, E11.319, E11.321, E11.329, E11.331, E11.339, E11.341, E11.349, E11.351, E11.359, E11.36, E11.39, E11.40, E11.41, E11.42, E11.43, E11.44, E11.49, E11.51, E11.52, E11.59, E11.610, E11.618, E11.620, E11.621, E11.622, E11.628, E11.630, E11.638, E11.641, E11.649, E11.65, E11.69, E11.8, E11.9, E13.00, E13.01, E13.10, E13.11, E13.21, E13.22, E13.29, E13.311, E13.319, E13.321, E13.329, E13.331, E13.339, E13.341, E13.349, E13.351, E13.359, E13.36, E13.39, E13.40, E13.41, E13.42, E13.43, E13.44, E13.49, E13.51, E13.52, E13.59, E13.610, E13.618, E13.620, E13.621, E13.622, E13.628, E13.630, E13.638, E13.641, E13.649, E13.65, E13.69, E13.8, E13.9, O24.011, O24.012, O24.013, O24.019, O24.02, O24.03, O24.111, O24.112, O24.113, O24.119, O24.12, O24.13, O24.311, O24.312, O24.313, O24.319, O24.32, O24.33, O24.811, O24.812, O24.813, O24.819, O24.82, O24.83, O24.911, O24.912, O24.913, O24.919, O24.92, O24.93. |
| Gestational hypertension | **ICD9: 642.0*, 642.1*, 642.2*, 642.7*:**  642.0, 642.00, 642.01, 642.02, 642.03, 642.04, 642.1, 642.10, 642.11, 642.12, 642.13, 642.14, 642.2, 642.20, 642.21, 642.22, 642.23, 642.24, 642.7, 642.70, 642.71, 642.72, 642.73, 642.74  **ICD10: O10*, O11*:**O10, O10.0, O10.01, O10.011, O10.012, O10.013, O10.019, O10.02, O10.03, O10.1, O10.11, O10.111, O10.112, O10.113, O10.119, O10.12, O10.13, O10.2, O10.21, O10.211, O10.212, O10.213, O10.219, O10.22, O10.23, O10.3, O10.31, O10.311, O10.312, O10.313, O10.319, O10.32, O10.33, O10.4, O10.41, O10.411, O10.412, O10.413, O10.419, O10.42, O10.43, O10.9, O10.91, O10.911, O10.912, O10.913, O10.919, O10.92, O10.93, O11, O11.1, O11.2, O11.3, O11.4, O11.5, O11.9 |
| Gestational diabetes | ICD-9: 648.80, 648.81, 648.82, 648.83, 648.84  **ICD-10:** O24.4, O24.41, O24.410 , O24.414, O24.415, O24.419, O24.42, O24.420, O24.424, O24.425, O24.429, O24.43, O24.430, O24.434, O24.435, O24.439, O99.81, O99.810, O99.814, O99.815 |

| **Table S2** Hydroxychloroquine (HCQ) exposure and initiation throughout pregnancy among patients with SLE, presented as n(%). A fill indicates a fill start and end date with any overlap with the period of interest. | | | |
| --- | --- | --- | --- |
| **Exposure definition and time period** | **All pregnancies**  **N = 477** | **Nulliparous pregnancies**  **N = 191** | **Multiparous pregnancies**  **N = 286** |
| 0 HCQ fills in 3 months pre LMP through first trimester | 220 (46%) | 78 (41%) | 142 (50%) |
| ≥1 HCQ fills in 3 months pre LMP through first trimester | 257 (54%) | 113 (59%) | 144 (50%) |
| ≥2 HCQ fills in 3 months pre LMP through first trimester | 179 (38%) | 85 (45%) | 94 (33%) |
| ≥2 HCQ fills in 3 months pre LMP through first trimester (continuous use through the entire exposure window with a 30-day allowable gap) | 89 (19%) | 48 (25%) | 41 (14%) |
| ≥1 HCQ fills in second trimester (with no prior fills in exposure window, does not consider fills in the 3^rd^ trimester) | 22 (4.6%) | 13 (6.8%) | 9 (3.1%) |
| ≥1 HCQ fills in third trimester (with no prior fills in exposure window or second trimester) | 8 (1.7%) | 6 (3.1%) | <5 |
| SLE = Systemic lupus erythematosus; LMP = last menstrual period | | | |

| **Table S3** Demographics and clinical characteristics for singleton deliveries (live and stillbirth) in patients with SLE by hydroxychloroquine (HCQ) exposure (≥ 1 fill vs 0 fills) and parity, presented as n(%) unless otherwise specified | | | | | |
| --- | --- | --- | --- | --- | --- |
| **Characteristics** | **All pregnancies** | **Nulliparous pregnancies** | | **Multiparous pregnancies** | |
|  |  | **HCQ +** | **HCQ -** | **HCQ +** | **HCQ -** |
|  | **n=477** | **n=113** | **n=78** | **n=144** | **n=142** |
| **Demographic characteristics** |  |  |  |  |  |
| Maternal age in years, median (IQR)^a^ | 32.40 [29.20, 35.40] | 30.50 [26.90, 34.10] | 30.95 [26.75, 33.38] | 32.80 [29.85, 36.12] | 33.90 [30.85, 36.00] |
| Race/ethnicity |  |  |  |  |  |
| *Asian* | 130 (27.3) | 37 ( 32.7) | 22 (28.2) | 40 (27.8) | 31 (21.8) |
| *Black* | 51 (10.7) | 15 ( 13.3) | 7 ( 9.0) | 15 (10.4) | 14 ( 9.9) |
| *Hispanic* | 117 (24.5) | 22 ( 19.5) | 22 (28.2) | 40 (27.8) | 33 (23.2) |
| *Other* ^b^ | 44 ( 9.2) | 12 ( 10.6) | 6 ( 7.7) | 13 ( 9.0) | 13 ( 9.2) |
| *White* | 135 (28.3) | 27 ( 23.9) | 21 (26.9) | 36 (25.0) | 51 (35.9) |
| Neighborhood deprivation index (residence at delivery) ^c^ |  |  |  |  |  |
| *Quintile 1 (Least deprived)* | 36 ( 9.9) | 7 ( 8.0) | <5 | 13 (13.1) | 12 (10.3) |
| *Quintile 2* | 70 (19.3) | 16 ( 18.4) | 16 (26.7) | 22 (22.2) | 16 (13.8) |
| *Quintile 3* | 90 (24.9) | 19 ( 21.8) | 16 (26.7) | 16 (16.2) | 39 (33.6) |
| *Quintile 4* | 90 (24.9) | 23 ( 26.4) | 12 (20.0) | 23 (23.2) | 32 (27.6) |
| *Quintile 5 (Most deprived)* | 76 (21.0) | 22 ( 25.3) | 12 (20.0) | 25 (25.3) | 17 (14.7) |
| Public insurance | 72 (15.2) | 17 ( 15.0) | 13 (16.7) | 22 (15.3) | 20 (14.3) |
| **Maternal morbidities and clinical characteristics** |  |  |  |  |  |
| History of lupus nephritis | 97 (20.3) | 26 ( 23.0) | 15 (19.2) | 32 (22.2) | 24 (16.9) |
| Positive aPL lab  ^d^ | 87 (19.6) | 24 ( 22.0) | 16 (22.2) | 24 (17.3) | 23 (18.4) |
| Pre-pregnancy hypertension | 78 (16.4) | 16 ( 14.2) | 17 (21.8) | 27 (18.8) | 18 (12.7) |
| Pre-pregnancy BMI, median (IQR) | 25.20 [22.00, 30.22] | 24.90 [21.80, 29.70] | 25.30 [22.15, 28.60] | 25.80 [22.00, 31.50] | 25.00 [21.95, 29.72] |
| **Pregnancy complications** |  |  |  |  |  |
| Gestational hypertension | 123 (25.8) | 39 ( 34.5) | 22 (28.2) | 30 (20.8) | 32 (22.5) |
| Preeclampsia | 90 (18.9) | 30 ( 26.5) | 16 (20.5) | 22 (15.3) | 22 (15.5) |
| Early onset preeclampsia (<34 weeks GA) | 28 ( 5.9) | 7 ( 6.2) | 6 ( 7.7) | 9 ( 6.2) | 6 ( 4.2) |
| PPROM | 23 ( 4.8) | 6 ( 5.3) | 3 ( 3.8) | 9 ( 6.2) | 5 ( 3.5) |
| Gestational diabetes | 36 (7.5) | 8 ( 7.1) | 3 ( 3.8) | 11 ( 7.6) | 14 ( 9.9) |
| **Prior pregnancy history** |  |  |  |  |  |
| Prior preterm (<37 weeks GA) |  |  |  | 44 (32.8) | 43 (33.6) |
| Prior preeclampsia |  |  |  | 34 (25.4) | 37 (28.9) |
| **Medications** |  |  |  |  |  |
| Corticosteroids ^e^ | 114 (23.9) | 40 ( 35.4) | 13 (16.7) | 45 (31.2) | 16 (11.3) |
| Azathioprine ^f^ | 45 (9.4) | 21 ( 18.6) | 7 (9.0) | 15 (10.4) | 7 (4.9) |
| Heparin ^f^ | 37 ( 7.8) | 11 ( 9.7) | 6 ( 7.7) | 12 ( 8.3) | 8 ( 5.6) |
| Labetalol ^f^ | 60 (12.6) | 19 ( 16.8) | 12 (15.4) | 18 (12.5) | 11 ( 7.7) |
| SLE = systemic lupus erythematosus, IQR = interquartile range, BMI = body mass index, aPL = antiphospholipid antibodies  ^a^ Maternal age at estimated date of last menstrual period (LMP)  ^b^ Other race and ethnicity category includes multiracial, Native American, Pacific Islander, and unknown  ^c^ Neighborhood deprivation index n=115 missing  ^d^ aPL n= 32 missing  ^e^ 6 months pre-LMP through LMP  ^f^ Pregnancy onset through delivery | | | | | |

| **Table S4** Demographics and clinical characteristics for singleton deliveries (live and stillbirth) in patients with SLE by continuous hydroxychloroquine (HCQ) exposure (≥ 2 fill vs 0 fills, gap in fills <30 days) and parity, presented as n(%) unless otherwise specified | | | | | |
| --- | --- | --- | --- | --- | --- |
| **Characteristics** | **All pregnancies** | **Nulliparous pregnancies** | | **Multiparous pregnancies** | |
|  |  | **HCQ +** | **HCQ -** | **HCQ +** | **HCQ -** |
|  | **n=309** | **n=48** | **n=78** | **n=41** | **n=142** |
| **Demographic characteristics** |  |  |  |  |  |
| Maternal age in years, median (IQR)^a^ | 32.70 [30.00, 35.60] | 32.45 [29.87, 36.12] | 30.95 [26.75, 33.38] | 32.70 [30.40, 35.10] | 33.90 [30.85, 36.00] |
| Race/ethnicity |  |  |  |  |  |
| *Asian* | 90 (29.1) | 22 ( 45.8) | 22 ( 28.2) | 15 ( 36.6) | 31 (21.8) |
| *Black* | 31 (10.0) | <5 | 7 ( 9.0) | 6 ( 14.6) | 14 ( 9.9) |
| *Hispanic* | 71 (23.0) | 9 ( 18.8) | 22 ( 28.2) | 7 ( 17.1) | 33 (23.2) |
| *Other* ^b^ | 26 ( 8.4) | <5 | 6 ( 7.7) | <5 | 13 ( 9.2) |
| *White* | 91 (29.4) | 9 ( 18.8) | 21 ( 26.9) | 10 ( 24.4) | 51 (35.9) |
| Neighborhood deprivation index (residence at delivery) ^c^ |  |  |  |  |  |
| *Quintile 1 (Least deprived)* | 24 ( 9.9) | <5 | <5 | <5 | 12 (10.3) |
| *Quintile 2* | 45 (18.5) | 7 ( 18.4) | 16 (26.7) | 6 ( 20.7) | 16 (13.8) |
| *Quintile 3* | 73 (30.0) | 13 ( 34.2) | 16 (26.7) | 5 ( 17.2) | 39 (33.6) |
| *Quintile 4* | 59 (24.3) | 9 ( 23.7) | 12 (20.0) | 6 ( 20.7) | 32 (27.6) |
| *Quintile 5 (Most deprived)* | 42 (17.3) | 5 ( 13.2) | 12 (20.0) | 8 ( 27.6) | 17 (14.7) |
| Public insurance | 39 (12.7) | 4 ( 8.3) | 13 (16.7) | 2 ( 4.9) | 20 (14.3) |
| **Maternal morbidities and clinical characteristics** |  |  |  |  |  |
| History of lupus nephritis | 60 (19.4) | 11 ( 22.9) | 15 (19.2) | 10 ( 24.4) | 24 (16.9) |
| Positive aPL lab ^d^ | 55 (19.3) | 11 ( 22.9) | 16 (22.2) | 5 ( 12.5) | 23 (18.4) |
| Pre-pregnancy hypertension | 49 (15.9) | 5 ( 10.4) | 17 (21.8) | 9 ( 22.0) | 18 (12.7) |
| Pre-pregnancy BMI, median (IQR) | 25.25 [22.00, 29.60] | 25.45 [21.98, 28.97] | 25.30 [22.15, 28.60] | 26.10 [22.90, 32.30] | 25.00 [21.95, 29.72] |
| **Pregnancy complications** |  |  |  |  |  |
| Gestational hypertension | 82 (26.5) | 20 ( 41.7) | 22 (28.2) | 8 ( 19.5) | 32 (22.5) |
| Preeclampsia | 55 (17.8) | 11 ( 22.9) | 16 ( 20.5) | 6 ( 14.6) | 22 (15.5) |
| Early onset preeclampsia (<34 weeks GA) | 18 ( 5.8) | <5 | 6 ( 7.7) | <5 | 6 ( 4.2) |
| PPROM | 16 ( 5.2) | <5 | <5 | 5 ( 12.2) | 5 ( 3.5) |
| Gestational diabetes | 23 ( 7.4) | <5 | <5 | <5 | 14 ( 9.9) |
| **Prior pregnancy history** |  |  |  |  |  |
| Prior preterm (<37 weeks GA) |  | 0 | 0 | 14 ( 35.9) | 43 (33.6) |
| Prior preeclampsia |  | 0 | 0 | 7 ( 17.9) | 37 (28.9) |
| **Medications** |  |  |  |  |  |
| Corticosteroids ^e^ | 62 (20.1) | 19 ( 39.6) | 13 (16.7) | 14 ( 34.1) | 16 (11.3) |
| Azathioprine ^f^ | 22 ( 7.1) | 10 ( 20.8) | 7 (9.0) | <5 | 7 (4.9) |
| Heparin ^f^ | 21 ( 6.8) | 5 ( 10.4) | 6 ( 7.7) | <5 | 8 ( 5.6) |
| Labetalol ^f^ | 36 (11.7) | 6 ( 12.5) | 12 (15.4) | 7 ( 17.1) | 11 ( 7.7) |
| SLE = systemic lupus erythematosus, IQR = interquartile range, BMI = body mass index, aPL = antiphospholipid antibodies  ^a^ Maternal age at estimated date of last menstrual period (LMP)  ^b^ Other race and ethnicity category includes multiracial, Native American, Pacific Islander, and unknown  ^c^ Neighborhood deprivation index n=66  missing  ^d^ aPL n=24 missing  ^e^ 6 months pre-LMP through LMP  ^f^ Pregnancy onset through delivery | | | | | |

| **Table S5** Pregnancy delivery outcomes and features by hydroxychloroquine (HCQ) exposure (≥ 1 fill vs 0 fills) and parity, presented as n(%) unless otherwise specified | | | | | |
| --- | --- | --- | --- | --- | --- |
| **Characteristics** | **All pregnancies** | **Nulliparous pregnancies** | | **Multiparous pregnancies** | |
|  |  | **HCQ+** | **HCQ-** | **HCQ+** | **HCQ-** |
|  | **n=477** | **n=113** | **n=78** | **n=144** | **n=142** |
| **Delivery outcomes & features** |  |  |  |  |  |
| Gestational age at delivery, median (IQR) | 38.00 [37.00, 39.00] | 38.00 [37.00, 39.00] | 38.00 [37.00, 39.00] | 38.00 [37.00, 39.00] | 39.00 [37.00, 39.00] |
| Gestational age at delivery among preterm deliveries, median (IQR)^a^ | 34.50 [32.50, 35.00] | 34.50 [32.50, 35.00] | 34.00 [30.00, 34.00] | 35.00 [29.50, 35.75] | 34.00 [33.00, 36.00] |
| Preterm (<37 weeks GA) | 98 (20.5) | 22 ( 19.5) | 13 (16.7) | 30 (20.8) | 33 (23.2) |
| Medically indicated ^a^ | 58 (59.2) | 12 (54.5) | 8 (61.5) | 14 (46.7) | 24 (72.7) |
| Spontaneous ^a^ | 35 (35.7) | 10 (45.5) | <5 | 15 (50.0) | 7 (21.2) |
| Extremely preterm (<28 weeks GA) ^a^ | 11 (11.2) | <5 | <5 | 5 (16.7) | <5 |
| Very preterm (28 to <32 weeks GA) ^a^ | 14 (14.3) | <5 | <5 | <5 | <5 |
| Moderately preterm (32 to <37 weeks GA) ^a^ | 73 (74.5) | 18 (81.8) | 8 (61.5) | 21 (70.0) | 26 (78.8) |
| Cesarean section ^b^ | 174 (38.2) | 43 ( 39.8) | 30 (40.5) | 52 (38.0) | 49 (36.0) |
| Stillbirths | 6 (1.2) | 0 ( 0.0) | <5 | <5 | <5 |
| IQR = interquartile range, GA = gestational age, PPROM = preterm premature rupture of membranes  ^a^ Denominator is preterm deliveries  ^b^ Cesarean section n = 22 missing | | | | | |

| **Table S6** Pregnancy delivery outcomes and features by continuous hydroxychloroquine (HCQ) exposure (≥ 2 fills vs 0 fills, gap in fills <30 days) and parity, presented as n(%) unless otherwise specified | | | | | |
| --- | --- | --- | --- | --- | --- |
| **Characteristics** | **All pregnancies** | **Nulliparous pregnancies** | | **Multiparous pregnancies** | |
|  |  | **HCQ+** | **HCQ-** | **HCQ+** | **HCQ-** |
|  | **n = 309** | **n=48** | **n=78** | **n=41** | **n=142** |
| **Delivery outcomes & features** |  |  |  |  |  |
| Gestational age at delivery, median (IQR) | 38.00 [37.00, 39.00] | 38.00 [37.00, 39.00] | 38.00 [37.00, 39.00] | 38.00 [37.00, 39.00] | 39.00 [37.00, 39.00] |
| Gestational age at delivery among preterm deliveries, median (IQR)^a^ | 34.00 [30.50, 35.75] | 34.50 [30.50, 35.00] | 34.00 [30.00, 34.00] | 35.00 [33.25, 35.75] | 34.00 [33.00, 36.00] |
| Preterm (<37 weeks GA) | 66 (21.4) | 10 ( 20.8) | 13 (16.7) | 10 ( 24.4) | 33 (23.2) |
| Medically indicated ^a^ | 43 (65.2) | 7 (70.0) | 8 (61.5) | <5 | 24 (72.7) |
| Spontaneous ^a^ | 19 (28.8) | <5 | <5 | 6 (60.0) | 7 (21.2) |
| Extremely preterm (<28 weeks GA) ^a^ | 7 (10.6) | <5 | <5 | <5 | <5 |
| Very preterm (28 to <32 weeks GA) ^a^ | 10 (15.2) | <5 | <5 | <5 | <5 |
| Moderately preterm (32 to <37 weeks GA) ^a^ | 49 (74.2) | 7 (70.0) | 8 (61.5) | 8 (80.0) | 26 (78.8) |
| Cesarean section ^b^ | 116 (37.5) | 18 ( 39.1) | 30 (40.5) | 19 ( 47.5) | 49 (36.0) |
| Stillbirths | <5 | 0 ( 0.0) | <5 | 0 ( 0.0) | <5 |
| IQR = interquartile range, GA = gestational age, PPROM = preterm premature rupture of membranes  ^a^ Denominator is preterm deliveries  ^b^ Cesarean section n = 13 missing | | | | | |

| **Table S7** The association between preterm birth and early pregnancy hydroxychloroquine (HCQ) exposure defined as ≥ 1 fills vs 0 fills and continuous use (≥ 2 fills vs 0 fills, gap in fills <30 days) estimated by modified poisson regression among patients with SLE stratified by parity | | | | | | | | | |
| --- | --- | --- | --- | --- | --- | --- | --- | --- | --- |
|  | **≥ 1 HCQ fills vs 0 fills** | | | |  | **Continuous use (≥ 2 HCQ fills vs 0 fills, gap <30 days)** | | | |
|  | **Nulliparous pregnancies** | | **Multiparous pregnancies** | |  | **Nulliparous pregnancies** | | **Multiparous pregnancies** | |
| ***Analyses*** | ***n*** ^a^ | ***RR (95% CI)*** | ***n*** ^a^ | ***RR (95% CI)*** |  | ***n*** ^a^ | ***RR (95% CI)*** | ***n*** ^a^ | ***RR (95% CI)*** |
| Crude | 181 | 1.03 (0.54, 1.95) | 258 | 0.89 (0.57, 1.39) |  | 113 | 1.04 (0.49, 2.23) | 141 | 0.98 (0.46, 2.12) |
| PS-adjusted ^b^ | 181 | 0.92 (0.46, 1.85) | 258 | 0.94 (0.58, 1.54) |  | 113 | 1.18 (0.50, 2.78) | 141 | 0.98 (0.45, 2.12) |
| **PS-adjusted stratified** | | | | |  |  | |  | |
| Pre-pregnancy hypertension | | | | |  |  | |  | |
| Yes | 31 | 1.11 (0.49, 2.51) | 41 | 1.19 (0.54, 2.62) |  | 18 | 1.25 (0.48, 3.25) | 23 | 1.49 (0.52, 4.26) |
| No | 150 | 0.92 (0.34, 2.49) | 217 | 0.79 (0.44, 1.41) |  | 95 | 1.16 (0.34, 4.03) | 118 | 0.63 (0.20, 1.97) |
| History of Lupus Nephritis | | | | |  |  | |  | |
| Yes | 37 | 1.28 (0.36, 4.57) | 52 | 0.98 (0.65, 1.47) |  | 25 | 1.81 (0.62, 5.28) | 27 | 1.82 (0.22, 14.97) |
| No | 144 | 0.92 (0.40, 2.12) | 206 | 0.93 (0.52, 1.66) |  | 88 | 0.94 (0.28, 3.22) | 114 | 0.80 (0.30, 2.13) |
| aPL positive | | | | |  |  | |  | |
| Yes | 39 ^c^ | 1.28 (0.37, 4.50) | 46 ^d^ | 0.99 (0.21, 4.63) |  | 25 | 1.84 (0.38, 9.03) | 23 ^e^ | NA^f^ |
| No | 132 ^c^ | 0.90 (0.40, 2.02) | 196 ^d^ | 0.91 (0.54, 1.55) |  | 88 | 1.00 (0.37, 2.69) | 114 ^e^ | 1.07 (0.45, 2.53) |
| Corticosteroid use during pregnancy | | | | |  |  | |  | |
| Yes | 47 | 0.70 (0.26, 1.91) | 50 | 1.36 (0.51, 3.59) |  | 29 | 0.90 (0.35, 2.34) | 19 | 2.22 (0.64, 7.64) |
| No | 134 | 1.12 (0.46, 2.74) | 208 | 0.89 (0.48, 1.62) |  | 84 | 1.38 (0.49, 3.90) | 122 | 0.77 (0.25, 2.35) |
| SLE = systemic lupus erythematosus, RR = risk ratio CI = confidence interval, PS = propensity score, NA = not applicable  ^a^ Sample size after trimming dataset based on area of common support  ^b^ The propensity score included covariates maternal age, pre-pregnancy BMI, maternal race/ethnicity, neighborhood deprivation index, diabetes, pre-pregnancy hypertension, history of lupus nephritis, pre-pregnancy corticosteroid use, azathioprine use during pregnancy, and aPL positive status. Prior preterm birth and prior preeclampsia were included for multiparous pregnancies.  ^c^ Missing aPL positive status for 10 nulliparous pregnancies  ^d^ Missing aPL positive status for 16 multiparous pregnancies  ^e^ Missing aPL positive status for 4 multiparous pregnancies  ^f^ Unable to calculate due to positivity violation: 0 preterm births with HCQ exposure | | | | | | | | | |

| **Table S8** The association between time to preterm delivery and early pregnancy hydroxychloroquine (HCQ) exposure defined as ≥ 1 fills vs 0 fills and continuous use (≥ 2 fills vs 0 fills, gap in fills <30 days) estimated by cox regression among patients with SLE stratified by parity. | | | | | | | | | |
| --- | --- | --- | --- | --- | --- | --- | --- | --- | --- |
|  | **≥ 1 HCQ fills vs 0 fills** | | | |  | **Continuous use (≥ 2 HCQ fills vs 0 fills, gap <30 days)** | | | |
|  | **Nulliparous pregnancies** | | **Multiparous pregnancies** | |  | **Nulliparous pregnancies** | | **Multiparous pregnancies** | |
| ***Analyses*** | ***n*** ^a^ | ***HR (95% CI)*** | ***n*** ^a^ | ***HR (95% CI)*** |  | ***n*** ^a^ | ***HR (95% CI)*** | ***n*** ^a^ | ***HR (95% CI)*** |
| Crude | 181 | 1.01 (0.50, 2.04) | 258 | 0.87 (0.52, 1.47) |  | 113 | 1.04 (0.44, 2.43) | 141 | 0.96 (0.41, 2.24) |
| PS-adjusted ^b^ | 181 | 0.89 (0.42, 1.88) | 258 | 0.92 (0.52, 1.63) |  | 113 | 1.20 (0.47, 3.09) | 141 | 0.95 (0.38, 2.38) |
| **PS-adjusted stratified** | | | | |  |  | |  | |
| Pre-pregnancy hypertension | | | | |  |  | |  | |
| Yes | 31 | 1.05 (0.35, 3.13) | 41 | 1.32 (0.46, 3.80) |  | 18 | 1.25 (0.31, 5.11) | 23 | 1.64 (0.40, 6.68) |
| No | 150 | 0.88 (0.32, 2.43) | 217 | 0.78 (0.39, 1.54) |  | 95 | 1.18 (0.34, 4.12) | 118 | 0.63 (0.17, 2.30) |
| History of Lupus Nephritis | | | | |  |  | |  | |
| Yes | 37 | 1.43 (0.31, 6.70) | 52 | 0.94 (0.48, 1.84) |  | 25 | 1.90 (0.40, 8.99) | 27 | 1.34 (0.22, 8.09) |
| No | 144 | 0.86 (0.34, 2.17) | 206 | 0.90 (0.30, 2.68) |  | 88 | 0.94 (0.27, 3.25) | 114 | 0.80 (0.26, 2.51) |
| aPL positive | | | | |  |  | |  | |
| Yes | 39^c^ | 1.22 (0.26, 5.64) | 46^d^ | 0.49 (0.10, 2.41) |  | 25 | 1.72 (0.29, 10.1) | 23 | f |
| No | 132^c^ | 0.85 (0.35, 2.04) | 196^d^ | 0.93 (0.49, 1.77) |  | 88 | 0.97 (0.32, 2.93) | 114^e^ | 1.16 (0.42, 3.16) |
| Corticosteroid use during pregnancy | | | | |  |  | |  | |
| Yes | 47 | 0.61 (0.19, 2.00) | 50 | 1.06 (0.35, 3.25) |  | 29 | 0.86 (0.24, 3.14) | 19 | 2.05 (0.50, 8.37) |
| No | 134 | 1.12 (0.43, 2.93) | 208 | 0.88 (0.44, 1.76) |  | 84 | 1.40 (0.39, 4.97) | 122 | 0.76 (0.21, 2.72) |
| SLE = systemic lupus erythematosus, RR = risk ratio CI = confidence interval, PS = propensity score, NA = not applicable  ^a^ Sample size after trimming dataset based on area of common support  ^b^ The propensity score included covariates maternal age, pre-pregnancy BMI, maternal race/ethnicity, neighborhood deprivation index, diabetes, pre-pregnancy hypertension, history of lupus nephritis, pre-pregnancy corticosteroid use, azathioprine use during pregnancy, and aPL positive status. Prior preterm birth and prior preeclampsia were included for multiparous pregnancies.  ^c^ Missing aPL positive status for 10 nulliparous pregnancies  ^d^ Missing aPL positive status for 16 multiparous pregnancies  ^e^ Missing aPL positive status for 4 multiparous pregnancies  ^f^ Unable to calculate due to positivity violation: 0 preterm births with HCQ exposure | | | | | | | | | |
